# Supplementary material for: Lung Inflammatory Phenotype in Mice Deficient in Fibulin-2 and ADAMTS-12
Source: Int J Mol Sci. 2024 Feb 7;25(4):2024. doi: 10.3390/ijms25042024 (PMC10888546; doi:10.3390/ijms25042024)
Supplement: Supplementary file 1 [file ijms-25-02024-s001.zip › ijms-2826931-supplementary.pdf]

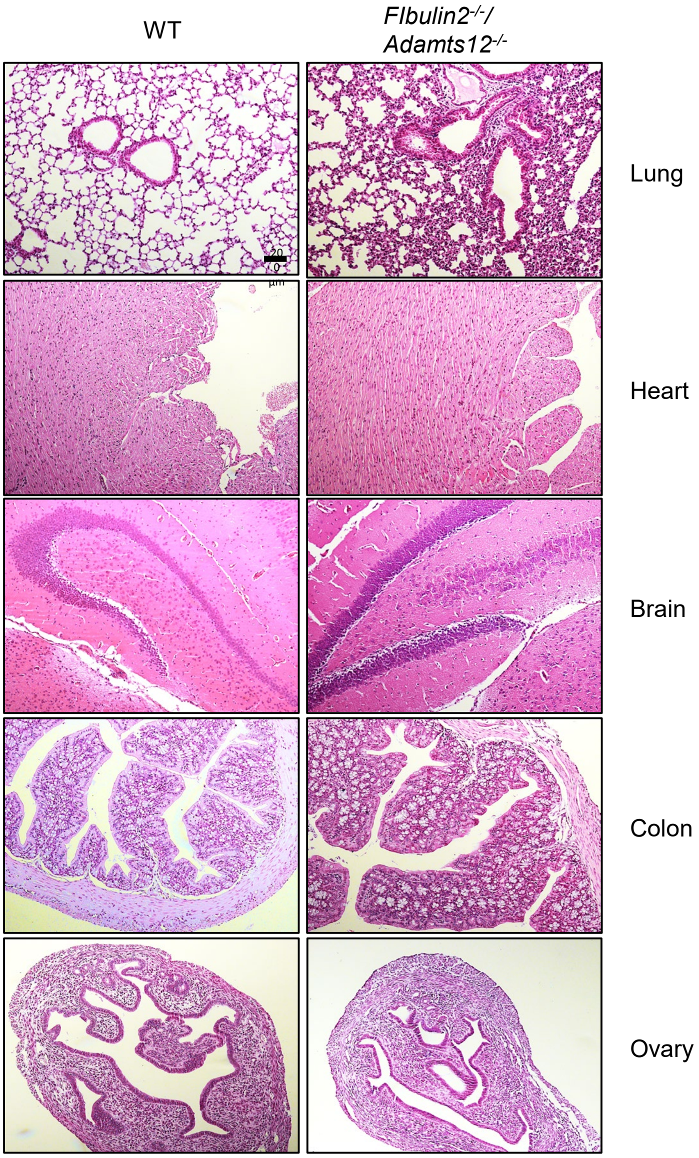

Figure S1. Haemotoxilin/eosin stained sections of the indicated tissues in WT and *fibulin2*$^{-/-}$/*Adamts12*$^{-/-}$ mice. Scale bar: 200μm

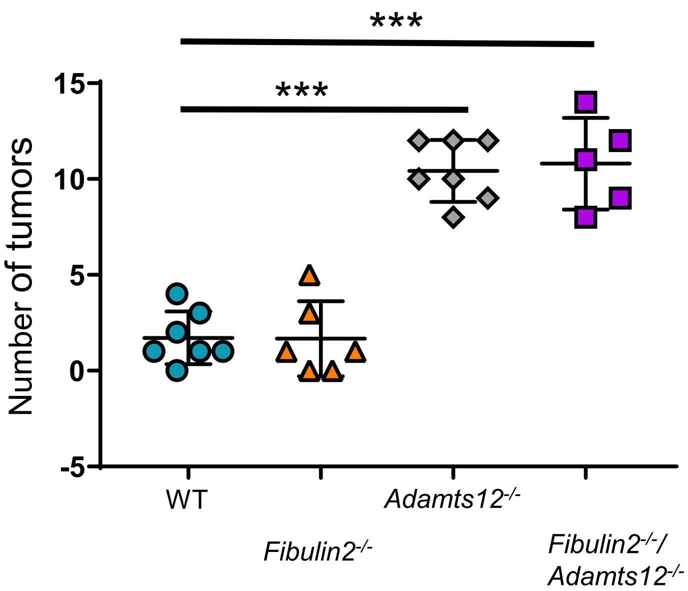

Figure S2. Lung cancer susceptibility of WT, *fibulin2$^{-/-}$, Adamts12$^{-/-}$* and *fibulin2$^{-/-}$/ADAMTS-12$^{-/-}$* mice.. Urethane lung carcinogenesis model in the indicated mice strains. The graph indicates the total number of tumors presented in the whole left lung of mice by tumor counting in haematoxilin/eosin stained sections.
